# Preoperative accuracy of diagnostic evaluation of urachal carcinoma

**DOI:** 10.1002/cam4.5648

**Published:** 2023-02-03

**Authors:** Chunjin Ke, Zhiquan Hu, Chunguang Yang

**Affiliations:** ^1^ Department of Urology, Tongji Hospital of Tongji Medical College Huazhong University of Science and Technology (HUST) Wuhan China

**Keywords:** cystoscopy, diagnosis, imaging, serum tumor markers, urachal carcinoma

## Abstract

**Background:**

We analyzed the clinical data of patients with urachal carcinoma (UrC) in order to strengthen urologists' understanding of UrC and improve preoperative diagnosis.

**Methods:**

The clinical data of 37 patients with UrC admitted to our hospital from October 2005 to April 2022 were retrospectively analyzed, and 40 patients with urothelial carcinoma (UCa) of bladder were enrolled as the control group. We compared and analyzed the imaging, cystoscopy and immunohistochemistry, serum tumor markers, fluorescence in situ hybridization (FISH) of UrC and bladder UCa for early diagnosis and evaluation of diagnostic accuracy.

**Results:**

A total of 37 patients with UrC were enrolled in this study, including 30 males and seven females, with a median age of 52.00 (44.50–63.50) years. Imaging and cystoscopy suggest that UrC grows primarily outside the bladder cavity and is found in the middle line of the dome or anterior wall of the bladder. There was a significant difference in tumor location between the UrC group and the UCa group (10.13 mm vs. −7.06 mm, *p* < 0.001). Immunohistochemistry revealed that CK20 and CDX‐2 were both diffusely and strongly positive. β‐catenin was strongly positive in cytoplasm and membrane, but negative in nuclear staining. Carcinoembryonic antigen (CEA) and carbohydrate antigen 72‐4 (CA724) expression levels were significantly higher in the UrC group than in the UCa group (*p* < 0.05). In the diagnosis of UrC, the area under the curve (AUC) of CEA combined with CA724 was the greatest. FISH's sensitivity in diagnosing UrC (5/7, 71.43%) was not significantly different from that of UCa (71.43% vs. 77.50%, *p* = 0.659). Imaging examination has the highest sensitivity and specificity among the accuracy evaluation of different diagnostic methods.

**Conclusions:**

Imaging and cystoscopy are the powerful diagnostic methods for UrC. Serum tumor markers may assist in diagnosis, prognosis, and monitoring. Positive urine FISH can easily misdiagnose UrC as UCa

## Graphical abstract

Preoperative diagnosis of urachal carcinoma: Imaging (A) , cystoscopy (B), tumor markers (C) and urine FISH (D).

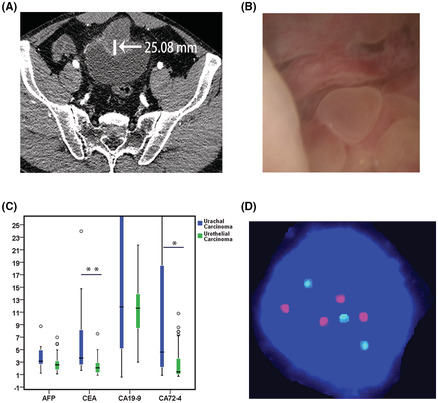

## 1 INTRODUCTION

During the embryonic period, the urachus is a tubular structure that extends from the front dome of the bladder to the umbilicus. The tubular structure disappears before birth and degenerates into the median umbilical ligament, which is located in the median umbilical fold.^1^, ^2^, ^3^ The urachus, also known as the median umbilical ligament, is an extraperitoneal structure with a length of about 2.0–15 cm and a diameter of 2–10 mm that is located in the loose connective tissue between the transverse abdominal fascia and the peritoneum (i.e., the Retzius space).^4^, ^5^ Urachal carcinoma (UrC) is an extremely rare and aggressive tumor originating from the urachus, with an annual incidence of about 1/5,000,000, accounting for 0.34% of bladder cancer.^6^, ^7^, ^8^ The median age of onset is 50–60 years old, and the most common histological type is adenocarcinoma (which can be divided into mucinous, enteric, and signet ring cell types), with urothelial carcinoma, neuroendocrine carcinoma, sarcoma, squamous cell carcinoma being uncommon.^9^, ^10^, ^11^, ^12^ Tumors are found throughout the urachus course in the Retzius space, with 90% located at the urachus junction, 6% in the middle, and 4% in the upper part of the urachus.^4^ Other abnormalities, such as cysts, fistula, diverticulum, infections and calcified stones, can occur within the Retzius gap, making differential diagnosis more difficult.^13^, ^14^ The disease has an insidious onset and is confined to the urachus in the early stage without clinical manifestations. When the lesion involves the bladder, the common symptoms are hematuria, followed by abdominal pain, urinary tract irritation, and umbilical secretions.^9^, ^15^ It is prone to metastasis and is often found to be at an advanced stage, with poor prognosis and serious threats to patients' lives,^15^ so early accurate diagnosis is particularly important.

As UrC is a rare disease, most of the articles published in the early stage are mainly case reports. In recent years, the research on UrC is relatively popular, but most of the research focuses on the treatment, survival prognosis, and molecular biology,^9^, ^10^, ^11^, ^15^, ^16^, ^17^, ^18^ while the research on the diagnostic accuracy of UrC is relatively few. Furthermore, most of the published studies focus on a certain diagnostic method, and do not compare and evaluate multiple diagnostic methods. There are many diagnostic methods for UrC diseases, such as imaging [ultrasound, computed tomography (CT), magnetic resonance imaging (MRI)], cystoscopy and biopsy, cytology, serum tumor markers, fluorescence in situ hybridization (FISH), etc. Ultrasound has the characteristics of non‐invasiveness, non‐radiative, and repetitiveness. MRI and CT can provide clearer and more accurate preoperative staging, detection of suspicious lymph nodes, and distant metastases. Compared with CT, MRI has no ionizing radiation and allows for more visualization of the anatomy.^6^, ^19^ Cystoscopy and biopsy of the bladder have high sensitivity and specificity, but this method has high medical costs and large trauma, and can only be used for patients with stage III (referring to Sheldon staging for UrC) and above. For patients with stage I and II, the effect is not effective, because early UrC is confined to the urachus and has not infringed the bladder, thus increasing the difficulty of the diagnosis of UrC.^9^ Serum tumor markers carcinoembryonic antigen (CEA), carbohydrate antigen 199 (CA199), and carbohydrate antigen 72‐4 (CA724) were originally markers of gastrointestinal and gynecological tumors, but were also significantly elevated in UrC, and their elevation was closely related to their staging and adverse prognosis.^20^, ^21^, ^22^ FISH is a molecular biological method approved by FDA for diagnosis and prognostic monitoring of urothelial carcinoma (UCa).^23^ However, Hu et al^24^ found that FISH also had a high positive rate in UrC in clinical practice, thus it was easy to misdiagnose UrC as UCa.

Mastering the advantages and disadvantages of different diagnosis methods is conducive to our precise diagnosis and treatment of UrC. The center retrospectively analyzed the relevant data of 37 patients admitted to Tongji Hospital Affiliated to Tongji Medical College of Huazhong University of Science and Technology from October 2005 to April 2022. We compared and analyzed the imaging, cystoscopy and immunohistochemistry, serum tumor markers, FISH, aiming to enhance urologists' understanding of UrC and improve the preoperative diagnostic level of UrC, so as to more comprehensively display the clinical characteristics of UrC and evaluate the accuracy of different diagnostic methods. It is summarized as follows:

## 2 METHODS

### 2.1 Study design and participants

With the approval of the Medical Ethics Committee of Tongji Hospital affiliated with Tongji Medical College, Huazhong University of Science and Technology (Approval No. TJ‐IRB20210521), we applied to the Department of Pathology to query the information of patients with UrC admitted to the Department of Urology from October 2005 to April 2022. Inclusion criteria: (1) Patients with UrC confirmed by imaging and pathology; (2) Not suffering from other types of tumors; (3) Detailed clinical records. In addition, 40 consecutive patients with bladder UCa in the same period, a certain time period and a single medical group were searched as a control group. Inclusion criteria: (1) Patients with UCa confirmed by pathology; (2) Not suffering from other types of tumors; (3) Specific clinical data such as imaging, cystoscopy, serum tumor markers, FISH, etc. The data of imaging, cystoscopy, tissue biopsy, serum tumor markers and FISH of UrC and bladder UCa were compared and analyzed to improve the accuracy of preoperative diagnosis.

### 2.2 Data Collection

The electronic medical record system at our institution was used to acquire the information. All subjects' demographics, clinical symptoms, laboratory data, imaging (CT/MRI), cystoscopy, FISH, histopathology, and immunohistochemistry were collected using standardized forms. The majority of the laboratory data consists of serum tumor indicators like alpha‐fetoprotein (AFP), CEA, CA199, and CA724.

### 2.3 Statistical analysis

Percentages were used to describe categorical variables, and the mean ± SD, or median with interquartile range were used to convey continuous variables. *T* tests were used to evaluate differences in continuous variables when they were normally distributed, otherwise, nonparametric tests were used. The Chi‐squared test was used to compare categorical variables. Logistic regression and ROC curves were established to evaluate the diagnostic efficacy of each serum tumor marker alone and in combination for UrC. All data analyses were performed using SPSS (version 24.0, IBM Corp.). Statistical significance was defined as a two sided of less than 0.05.

## 3 RESULTS

### 3.1 Clinical and demographic features of UrC

This study included 37 UrC patients, 30 males and 7 females, with a median age of 52.00 (44.50–63.50) years and an average age of 52.86 (25–81) years. Gross hematuria is the most common initial symptom, followed by bladder urinary tract irritation, abdominal pain, and lower abdominal mass. Some patients may have umbilical blood and purulent secretions. The mass of 34 (91.89%) patients with UrC was located in the middle of the dome or anterior wall of the bladder. The pathological findings of all 37 patients were adenocarcinoma. The histological type was divided into enteric adenocarcinoma [*n* = 17 (45.95%)], mucinous adenocarcinoma [*n* = 8 (21.62%)], signet ring cell carcinoma [*n* = 2 (5.41%)], and unclassified 10 cases. According to Sheldon staging, there were two (5.41%) cases in stage I‐II, 23 (62.16%) in stage IIIa, four (10.81%) in stage IIIb, two (5.41%) in stage IIIc, and five (13.51%) in stage IV. One case was transferred to our hospital after surgery from another hospital, and there is no specific staging data. (Table 1).

**TABLE 1:** Baseline characteristics of patients with urachal and urothelial carcinoma.

Parameters	Urachal carcinoma (*n* = 37)	Urothelial carcinoma (*n* = 40)		*p*
Sex, (*n*, %)				0.646
Male	30 (81.08%)	34 (85.00%)		
Female	7 (18.92%)	6 (15.00%)		
Median age, (interquartile range, years)	52.00 (44.50–63.50)	56.50 (51.00–65.75)		0.176
Initial symptoms, *n* (%)				0.737
Gross hematuria	27 (72.97%)	32 (80.00%)		
Lumps or pain in the lower abdomen	5 (13.51%)	1 (2.50%)		
Pain or discharge around the umbilicus	1 (2.70%)	0 (0)		
Urinary tract irritation	8 (21.62%)	9 (22.50%)		
Tumor location, *n* (%)				**<0.001**
Midline of apical or anterior wall	34 (91.89%)	0 (0)		
Midline of posterior wall	2 (5.41%)	1 (2.50%)		
Periumbilical or subumbilical	1 (2.70%)	0 (0)		
Side wall and triangle area	0 (0)	39 (97.50%)		
Cystoscopy accuracy^a^, *n* (%)				**0.013**
Consistent	11 (52.38%)	33 (82.50%)		
Inconsistent	10(47.62%)	7 (17.50%)		
Fluorescence in situ hybridization^b^, *n* (%)				0.659
Positive	5 (71.43%)	31 (77.50%)		
Negative	2 (28.57%)	9 (22.50%)		
Serum tumor markers, median ± quartile				
AFP, ng/mL	3.15 (2.26–5.21)	2.55 (1.79–3.18)		0.128
CEA, ng/mL	3.65 (2.29–11.47)	2.08 (1.30–2.94)		**0.001**
CA199, U/mL	11.86 (5.13–163.48)	11.65 (8.40–14.00)		0.741
CA724, U/mL	4.61 (1.52–29.02)	1.43 (1.05–3.64)		**0.038**
Immunohistochemical positive rate (%)				
CK‐7	55.00% (11/20)	69.57% (16/23)		0.361
CK‐20	90.00% (18/20)	47.83% (11/23)		**0.004**
CDX‐2	78.95% (15/19)			
Villin	94.12% (16/17)			
β‐catenin	100% (3/3)			
GATA‐3	0 (0/10)	100% (29/29)		**<0.001**
PSA	0 (0/7)	0 (0/9)		
Ki‐67 > 47.5%	64.29% (9/14)	34.48% (10/29)		0.102
Sheldon/TNM, *n* (%)				
I‐II	2 (5.41%)	Ta	12 (30.00%)	
IIIa	23 (62.16%)	T1	23 (57.50%)	
IIIb	4 (10.81%)	T2	3 (7.50%)	
IIIc	2 (5.41%)	T3	2 (5.00%)	
IV	5 (13.51%)	T4	0 (0)	
Histologic type, *n* (%)				
Mucinous features	8 (21.62%)			
Enteric features	17 (45.95%)			
Signet ring cell features	2 (5.41%)			

Abbreviations: AFP, alpha‐fetoprotein; CEA, carcinoembryonic antigen; CA199, carbohydrate antigen199; CA724, carbohydrate antigen 72‐4; CK‐7, cytokeratin‐7; CK‐20, cytokeratin‐20; CDX‐2, caudal type homeobox transcription factor 2; PSA, prostate specific antigen.

^a^ Only 21 patients with urachal carcinoma underwent cystoscopy.

^b^ Fluorescence in situ hybridization in seven patients with urachal carcinoma.

Bold of “< 0.001” means statistical significance difference between two groups.

### 3.2 Comparative analysis of imaging (CT/MRI) between the two groups

**FIGURE 1:**
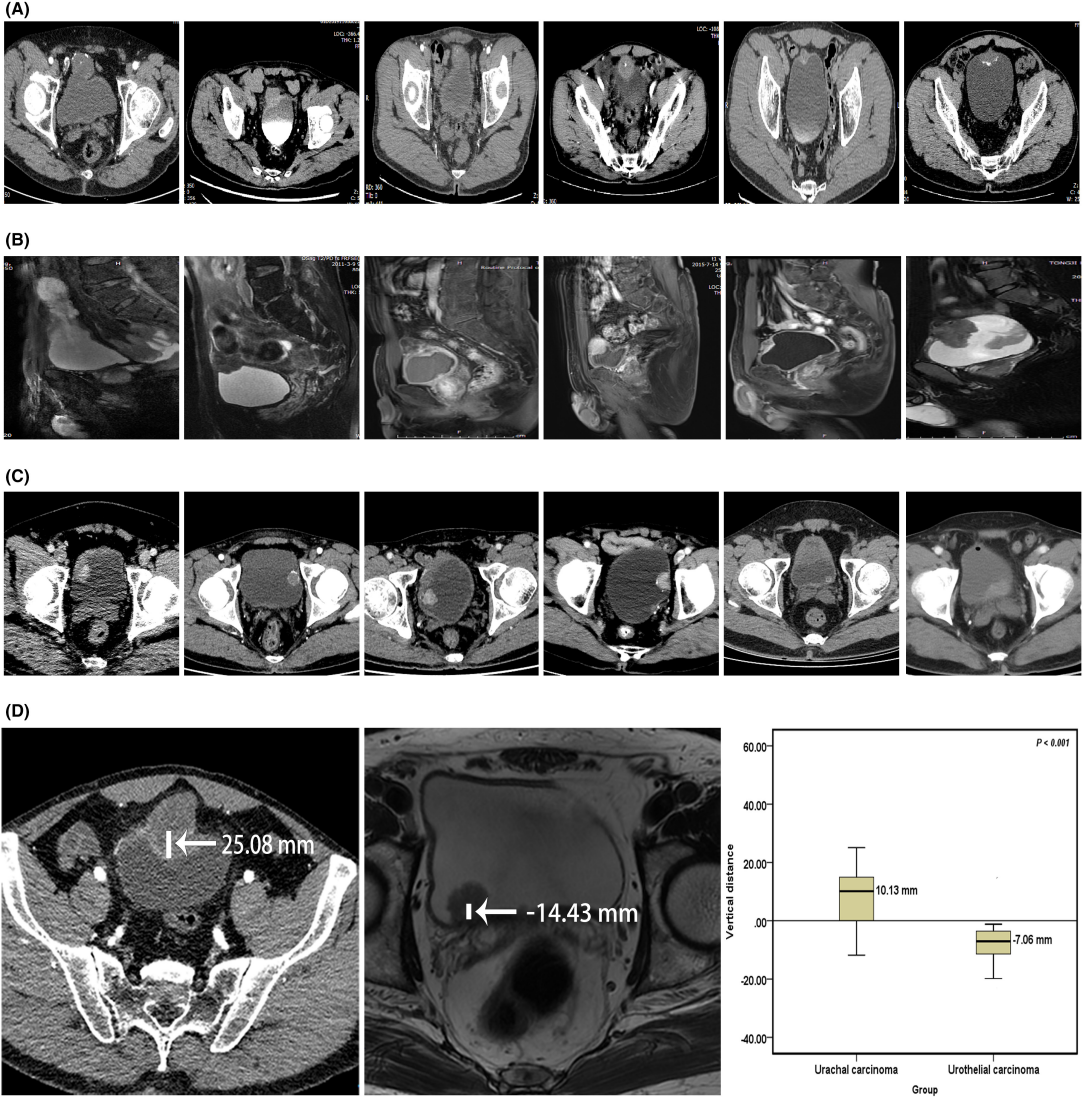
Comparison of imaging features between urachal carcinoma and urothelial carcinoma. (A) CT of urachal carcinoma. (B) MRI of urachal carcinoma. (C) CT of urothelial carcinoma. (D) Differences in tumor growth locations. CT, computed tomography; MRI, magnetic resonance imaging.

UrC is mostly cystic or solid, and the parenchymal component enhances with contrast. The central or peripheral part of the mass is mostly punctate, patchy or annular calcification (Figure 1A). Thin‐slice sagittal reconstruction images can clearly show the relationship between the tumor, urachus, and bladder (Figure 1B). The vertical distance between the center of the mass and the bladder wall was measured. The mass in the bladder cavity was marked as “‐” and the mass outside the bladder cavity was marked as “+”. If the mass invaded the bladder wall and completely fused with it, so that the center fell on the bladder wall, it was marked as “0”. According to the imaging features and data analysis (Figure 1D), there was a significant difference in the tumor location between the UrC and UCa groups (10.13 mm vs. −7.06 mm, *p* < 0.001). UrC are mostly located in the middle line of the dome or anterior wall of the bladder. They mainly grow outside the bladder cavity and often invade the bladder wall, resulting in thickening of the adjacent bladder wall and deformation of the bladder, and grow into the bladder cavity. However, bladder UCa is mostly located in the lateral wall of the bladder or trigone (Figure 1C), which can be multiple. The clinical symptoms appear earlier and are usually found in the early stage.

### 3.3 Comparative analysis of cystoscopy and immunohistochemistry between the two groups

**FIGURE 2:**
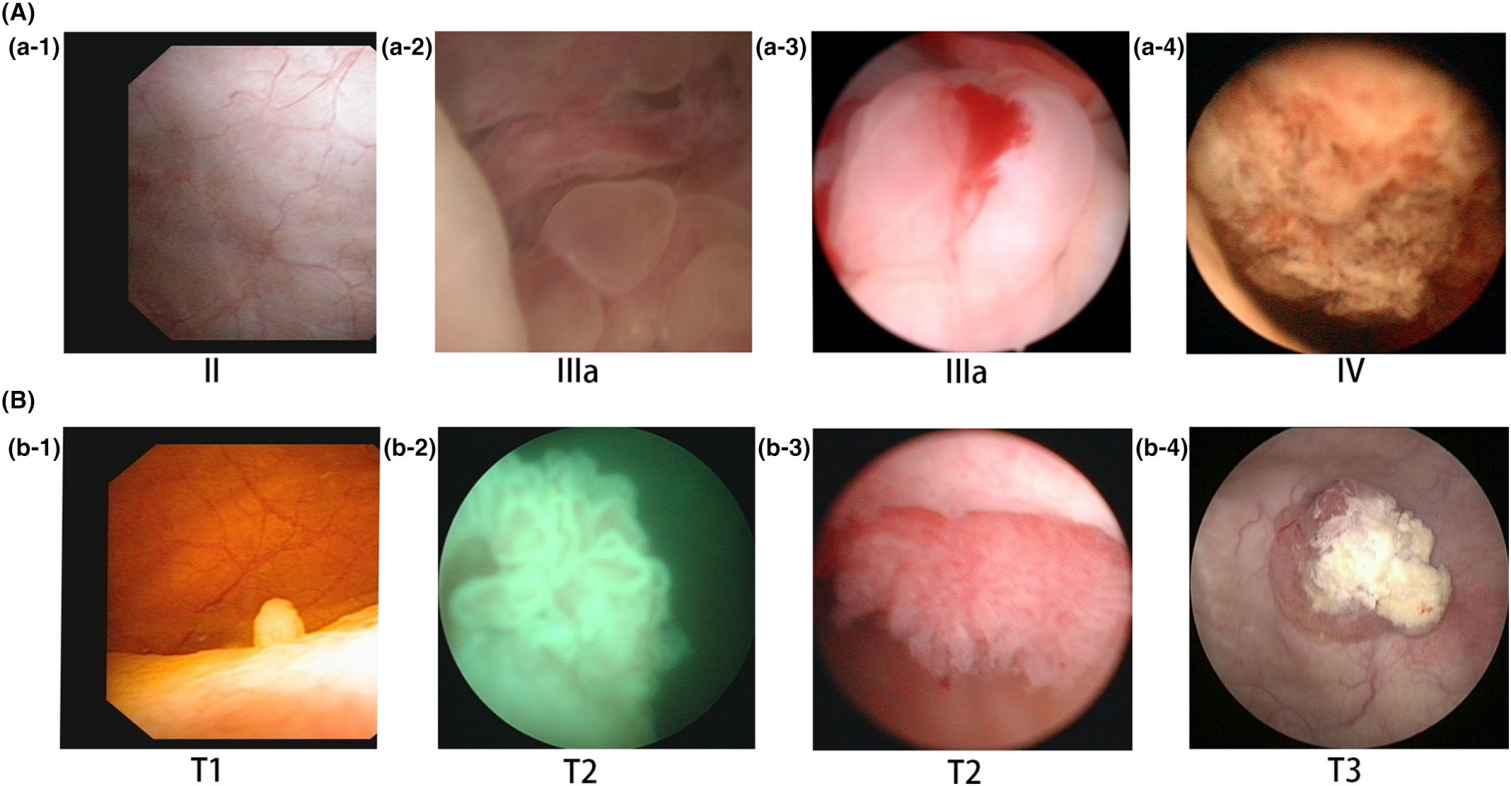
Cystoscopy comparison of urachal carcinoma and urothelial carcinoma. (A) Cystoscopy of urachal carcinoma; (B) cystoscopy of urothelial carcinoma.

**FIGURE 3:**
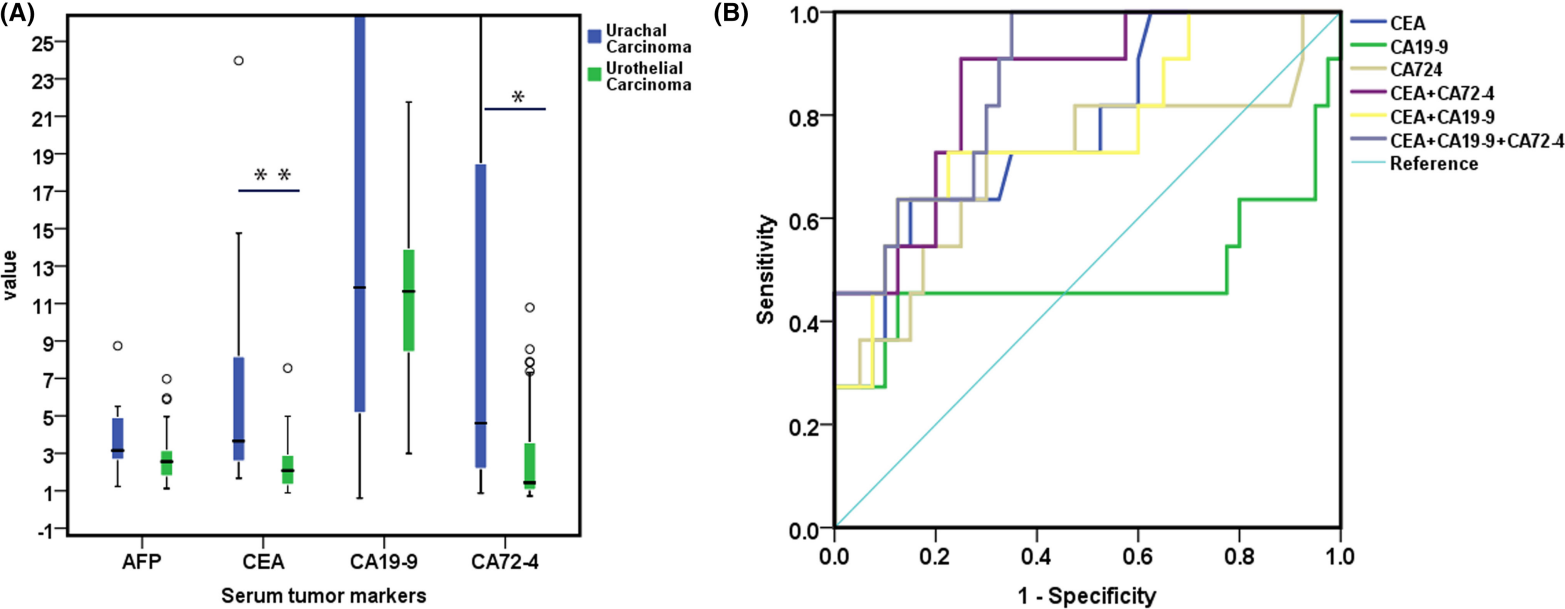
Diagnostic role of serum tumor markers for urachal carcinoma. (A) Difference analysis of serum tumor markers between urachal carcinoma and urothelial carcinoma, **p = 0.001, *p = 0.038. (B) ROC curve of serum tumor markers for diagnosis of urachal carcinoma by single and combined detection. AFP, alpha‐fetoprotein; CEA, carcinoembryonic antigen; CA199, carbohydrate antigen199; CA724, carbohydrate antigen 72‐4.

It can be seen from the cystoscopy in Figure 2 that: When the UrC does not break through the full thickness of the bladder wall, cystoscopy showed that the anterior top wall of the bladder is compressed and bulges into the cavity, and the bladder mucosa is intact or ulcerated (Figure 2A‐1). After breaking through the bladder wall, surrounding mucus components or new follicular organisms could be seen (Figure 2A‐2,A‐3). When the mass in the bladder cavity is large, the irregular hard gray mass with a broad base can be seen (Figure 2A‐4). In UCa, the early stage may be single or multiple, pink, villous or papillary (Figure 2B‐1). Villous lesions often merge into clusters, or are broadly basal‐like, with poor mobility, cauliflower‐like, and infiltrating growth (Figure 2B‐2,B‐3). Advanced UCa can break through the bladder wall, grow out of the bladder cavity, and invade surrounding tissues (Figure 2B‐4). However, the accuracy rate of cystoscopy in diagnosing UrC was only 52.38% (11/21), which was significantly lower than that of UCa (52.38% vs. 82.50%, *p* = 0.013) (Table 1). Therefore, cystoscopy has limitations in the diagnosis of UrC, which needs to be combined with imaging and other clinical data.

Immunohistochemical comparison: In UrC, CK20, and CDX‐2 can be diffusely strong positive. CK7 can have a positive rate of 55.00%. β‐catenin can be strongly positive in cytoplasm and cell membrane, but negative in nuclear staining. Ki‐67 > 47.5% accounted for 64.29%, but GATA‐3 and PSA were negative. The positive rate of GATA‐3 in UCa was 100%, and GATA‐3 and CK7 were strongly positive and diffusely positive (Table 1).

### 3.4 Comparative analysis of serum tumor markers between the two groups

The expression levels of CEA and CA724 in the UrC group were significantly higher than those in the UCa group (*p* < 0.05) (Table 1, Figure 3A). At the time of data collection, we also found that patients with distant metastatic UrC had higher CA724 abnormalities than the non‐metastatic group. When serum tumor markers were detected alone, CEA had the highest AUC (S = 0.773, 95%CI: 0.616–0.930, *p* = 0.006) in the diagnosis of UrC. When combined detection, the AUC of CEA combined with CA724 was the largest (S = 0.855, 95% CI: 0.735–0.974, *p* < 0.001) (Figure 3B).

### 3.5 Comparative analysis of FISH between the two groups

Only 8 of the 37 patients with UrC underwent urine UroVysion™ FISH test, but one was the result of detection after radical resection, so it was excluded. In the UrC group, urine FISH was positive in five cases and negative in two cases, and the sensitivity for diagnosing UrC was 71.43% (5/7). In the control group, urine FISH was positive in 31 cases and negative in nine cases, with a sensitivity of 77.50% (31/40). There was no significant difference in the diagnostic efficacy of urine FISH in UrC and UCa (71.43% vs. 77.50%, *p* = 0.659) (Table 1).

### 3.6 Diagnostic evaluation

According to Table 2, the sensitivity, specificity, and accuracy of imaging in preoperative diagnosis of UrC are relatively high. Serum tumor markers can assist in diagnosis, prognosis, and monitoring. Positive urine FISH can easily misdiagnose UrC as UCa.

**TABLE 2:** Preoperative accuracy of diagnostic evaluation of urachal carcinoma.

Parameter	No. Pts	Sensitivity	Specificity	PPV	NPV	Accuracy
CT	72	85.71%	81.82%	75.00%	90.00%	83.33%
MRI	72	96.77%	95.12%	93.75%	97.50%	95.83%
Cystoscopy	61	52.38%	82.50%	61.11%	76.74%	72.13%
Cytology^8^	102	29.41%	—	—	—	—
Serum tumor markers						
CEA > 6.83 ng/mL	53	30.77%	97.50%	80.00%	81.25%	81.13%
CA724 > 6.02 U/mL	51	36.36%	85.00%	40.00%	82.93%	74.51%
CA199 > 9.935 U/mL	53	53.85%	40.00%	22.58%	72.73%	43.40%
FISH	47	71.43%	22.50%	13.90%	81.82%	29.79%

*Note*: The control group was patients with urothelial carcinoma.

Abbreviations: CA199, carbohydrate antigen199; CA724, carbohydrate antigen 72‐4; CEA, carcinoembryonic antigen; CT, computed tomography; FISH, fluorescence in situ hybridization; MRI, magnetic resonance imaging; NPV, negative predictive value; PPV, positive predictive value; Pts, patients.

## 4 DISCUSSION

UrC is an extremely rare and malignant disease. Mastering the anatomy of urachus is the prerequisite for diagnosis of UrC. In this study, we specifically described the CT and MRI imaging features of UrC and evaluated its diagnostic accuracy. It was found that 91.89% of the UrC were located in the dome or anterior wall of the bladder, infiltrating the muscular layer or breaking through the bladder wall. Most of the masses were cystic or solid, and the parenchymal components were significantly enhanced with contrast, and the central or peripheral parts of the masses were mostly punctured, patchy or ring calcification, which was consistent with the results of many imaging studies related to UrC.^6^, ^8^, ^15^, ^19^, ^25^ Among various diagnostic methods, we found that MRI had the highest diagnostic accuracy, reaching 95.83%. Thin layer sagittal reconstruction image can clearly show the relationship between tumor, urachus and bladder, and provide more accurate clinical data for tumor staging and formulation of treatment plan.^19^ Other studies have shown that MRI can better distinguish the infection of urachus from UrC,^6^ so MRI is a no ‐invasive and valuable diagnosis method for precision diagnosis and treatment of UrC.

Cystoscopy is the most direct visual examination means for the diagnosis of UrC, which can detect whether the bladder is involved. In this study, it was found that the accuracy rate of cystoscopy in the diagnosis of UrC was only 52.38%, significantly lower than that of UCa (52.38% vs. 82.50%, *p* = 0.013). However, Tibor Szarvas et al.^8^ reported in a meta‐analysis of 1010 cases of UrC that the accuracy rate of cystoscopy biopsy reached 89%, and cytological diagnostic sensitivity was only 29%. This difference may be caused by the limited material for biopsy and the tumor's failure to break through the entire layer of the bladder wall. Immunohistochemistry indicated that both CK20 and CDX‐2 were diffuse positive. CK7 had a positive rate of 55.00%. β‐catenin was positive in cytoplasm and cell membrane, but negative in nuclear staining.^26^, ^27^ However, studies published in the *European Journal of Urology* show that there is a significant genomic and histological similarity between UrC and colorectal cancer,^28^, ^29^ so the diagnosis and treatment of UrC should be referred to as well as differentiated from colorectal cancer. Colorectal cancer is easily identified by colonoscopy and biopsy. It does not express high molecular weight cytokeratin 34βE12 and CK7, but it is nuclear positive for β‐catenin, which rarely occurs in UrC.^27^, ^29^ Primary adenocarcinoma of kidney, breast, stomach, endometrium, and other organs may metastasize to the bladder or urachus, but it is extremely rare.^30^ However, the author collected data on a case of gastric adenocarcinoma metastasized to the urachus.

Studies have confirmed that CEA, CA199, and CA724 are important monitoring markers of colorectal adenocarcinoma, lung adenocarcinoma, gastric adenocarcinoma, pancreatic cancer, and other adenocarcinomas, which are closely related to the development, treatment, and prognosis of the disease.^20^ The main histological type of UrC is adenocarcinoma, accounting for more than 90%, which has obvious similarities with colorectal adenocarcinoma in histommorphology.^29^, ^31^ Therefore, the application value of CEA, CA199, and CA724 in UrC is based on evidence. This study showed that CEA had the largest AUC when a single index was used as a diagnostic test, and its expression level was significantly higher than that of the bladder UCa. The AUC of CA199 was less than 0.5, indicating that CA199 had little significance in the diagnosis of UrC. The AUC of CEA and CA724 combined tests was the largest, both larger than that of single test and other combined tests, but the differences were not statistically significant, which may be related to the insufficient sample size in this study. When analyzing the data in this study, it was found that the expression level of tumor markers in patients with UrC was higher with the later stage, and the tumor markers could be significantly reduced after surgery or chemotherapy, which was related to treatment reactivity, and it was promising to be applied to the monitoring and prognosis evaluation of UrC. However, due to the small sample size and incomplete data, statistical difference analysis could not be made. Siefker‐Radtke et al.^32^ also found that CEA serum level increased in 59% of patients with UrC, and CEA also decreased due to chemotherapy, suggesting that CEA detection has potential application value in monitoring (or follow‐up) of UrC, which is consistent with the results of this study. Reis et al.^21^ and Zong et al^22^ found that CA724 increased significantly when UrC metastasis or recurrence occurred, which was consistent with the conclusion reached in this study that the positive detection rate of CA724 was significantly higher in the metastatic group than in the non‐metastatic group. Reis et al.^21^ also found that increased CEA levels were associated with worse median overall survival (*p* = 0.008) and median progression‐free survival (*p* = 0.009). In conclusion, serum tumor markers have certain reference value for diagnosis, staging, progression, treatment response, and prognosis of UrC.

The positive manifestations of FISH in UrC are unintentionally discovered by the team in clinical practice. Previous published studies of our team showed that there was no statistical significance in the diagnostic efficacy of FISH in UrC and UCa (71.43% vs. 77.50%, *p* = 0.659), and histological FISH and urine FISH were used to verify each other.^24^ However, the Food and Drug Administration (FDA) approved UroVysion™ probes (chromosome 3, 7, 17 combined with 9p21 probe) for urine detection and postoperative recurrence monitoring in patients with suspected bladder cancer in 2001 and 2005, respectively.^33^ Hence, the application of FISH detection in patients with hematuria may easily misdiagnose UrC as UCa, thus leading to the formulation of wrong treatment plan. The following analysis was made for the positive performance of UroVysion™ FISH in urine samples of UrC. The Beijing Jinpujia Medical Technology Co., Ltd.‐provided centromere probe and site‐specific recognition probe, comprised of the two combinations CSP3 (green)/CSP7 (red) and GLP p16 (red)/CSP17 (green), are the FISH DNA probes employed at our hospital. If the tumor cells have chromosome 3, 7, 17 aberrations or (and) deletion of the GLP p16 gene locus, and the diseased cells can be shed in sufficient quantities into the urine, the urine FISH may be positive. Sequence variation was revealed in *TP53*, *PIK3CA*, *BRAF*, *NRAS*, *KRAS*, *FGFR1*, *MET*, and *PDGFRA* in a gene sequencing analysis of 70 instances of UrC, while gene amplification was discovered in EGFR, ERBB2, and MET. These genes can produce positive FISH results because they are located on the chromosomes 17p13, 3p21, 7p12, and 17p21.^34^

This study also has some shortcomings. Due to the low incidence of UrC, there are few data on serum tumor markers and FISH, and the conclusion lacks the support of multicenter big data. Nevertheless, this does not affect the reliability of our results, because this study was compared with existing studies and no obvious bias was found.

Although there are certain limitations, this study presents the clinical characteristics of UrC in a more comprehensive way through different diagnostic methods and evaluates the diagnostic accuracy, enabling urological surgeons to have a more systematic understanding of the diagnosis of UrC [①Imaging (CT or MRI): identify structures, infer properties; ②Cystoscope + biopsy: low accuracy of biopsy in exploring bladder involvement; ③FISH: to avoid misdiagnosis; ④Cytology: low sensitivity, no differential diagnosis; ⑤Serum tumor markers: assist in diagnosis and prognosis], so as to realize the accurate diagnosis and treatment of UrC.

## 5 CONCLUSIONS

Familiarity with the anatomy of the urachus is a prerequisite for the diagnosis of UrC. Imaging and cystoscopy are powerful diagnostic methods for UrC. Serum tumor markers may assist in diagnosis, prognosis, and monitoring. Positive urine FISH is easy to misdiagnose UrC as UCa, and is not reliable in the diagnosis of UrC. Mastering the advantages and disadvantages of different diagnosis methods is conducive to our precise diagnosis and treatment of UrC.

## ETHICAL APPROVAL AND CONSENT TO PARTICIPATE

This study was reviewed and approved by the Medical Ethics Committee of the Tongji Hospital of Huazhong University of Science and Technology (IRB ID: TJ‐IRB20210521) and individual consent for this retrospective analysis was waived.

## HUMAN ETHICS

All procedures performed in studies involving human participants were in accordance with the ethical standards of the Lifespan institutional research committee and with the 1964 Helsinki declaration and its later amendments or comparable ethical standards.

## CONSENT FOR PUBLICATION

On behalf of all authors, consent for publication if accepted. The copyright to this article is transferred to Huazhong University of Science and Technology and Springer effective if and when the article is accepted for publication.

## AUTHOR CONTRIBUTIONS

**Chunjin Ke:** Conceptualization (equal); data curation (equal); formal analysis (equal); writing – original draft (equal). **Zhiquan Hu:** Conceptualization (equal); funding acquisition (supporting); project administration (lead); supervision (lead). **Chunguang Yang:** Funding acquisition (lead); project administration (lead); writing – review and editing (lead).

## FUNDING INFORMATION

This work was supported by the National Natural Science Foundation of China (No. 81702989) and Sanming Project of Medicine in Shenzhen (No.SZSM202111003). The authors would like to thank Professor Zhangqun Ye (Department of Urology, Tongji Hospital, Tongji Medical College, Huazhong University of Science and Technology) for Funding acquisition.

## CONFLICT OF INTEREST STATEMENT

The authors declare no conflict of interest.

## Data Availability

With the permission of the corresponding authors, the raw data without names and identifiers are available upon reasonable request. Data can be provided after publication of this study. Once the data are approved to be made public, the researchers will provide an email address for communication. The corresponding authors will make a decision based on the research objectives and plan provided
